# Comparative quantitative proteomics of *prochlorococcus *ecotypes to a decrease in environmental phosphate concentrations

**DOI:** 10.1186/2046-9063-8-7

**Published:** 2012-03-19

**Authors:** Matthew A Fuszard, Phillip C Wright, Catherine A Biggs

**Affiliations:** 1 ChELSI Institute, Department of Chemical and Biological Engineering, University of Sheffield, Mappin Street, Sheffield S1 3JD, UK

**Keywords:** *Prochlorococcus*, PstS, PhoA, PhoE, Growth, Phosphate

## Abstract

**Background:**

The well-lit surface waters of oligotrophic gyres significantly contribute to global primary production. Marine cyanobacteria of the genus *Prochlorococcus *are a major fraction of photosynthetic organisms within these areas. Labile phosphate is considered a limiting nutrient in some oligotrophic regions such as the Caribbean Sea, and as such it is crucial to understand the physiological response of primary producers such as *Prochlorococcus *to fluctuations in the availability of this critical nutrient.

**Results:**

*Prochlorococcus *strains representing both high light (HL) (MIT9312) and low light (LL) (NATL2A and SS120) ecotypes were grown identically in phosphate depleted media (10 μM P_i_). The three strains displayed marked differences in cellular protein expression, as determined by high throughput large scale quantitative proteomic analysis. The only strain to demonstrate a significantly different growth rate under reduced phosphate conditions was MIT9312. Additionally, there was a significant increase in phosphate-related proteins such as PhoE (> 15 fold increase) and a depression of the Rubisco protein RbcL abundance in this strain, whereas there appeared to be no significant change within the LL strain SS120.

**Conclusions:**

This differential response between ecotypes highlights the relative importance of phosphate availability to each strain and from these results we draw the conclusion that the expression of phosphate acquisition mechanisms are activated at strain specific phosphate concentrations.

## Background

Within marine oligotrophic systems, such as central subtropical gyres, orthophosphate (P_i_) is a crucial macronutrient governing microbial population densities, particularly within the well-lit surface waters of the euphotic zone [1-3]. The principal photosynthetic organism numerically dominating these areas is *Prochlorococcus*, which is estimated to represent about 50% of all photosynthetic activity within them [4,5]. *Prochlorococcus *has been broadly delineated into two clades, or ecotypes, high light (HL) and low light (LL) based upon the ratios of divinylchlorophyll*a *and *b *within their light harvesting apparatuses and as such their assumed depth within the water column [6,7]. Further clade subdivisions have been implemented through phylogenetic analyses of 16S rRNA sequences [8]. As a taxon, *Prochlorococcus *is characterised by its small size (~ 1 μm^3^), and significantly reduced genomes which ranges from 1.64 Mbps (the HL strain MIT9301) to 2.68 Mbps (the LL strain MIT9303) [9]. This diminished volume and genome is hypothesised to be the result of an accelerated evolutionary process adapting to reduced phosphorus in its environment [10,11]. Indeed, *Prochlorococcus *is known to replace phospholipids in its membranes with sulpholipids, which dramatically reduce its P_i _requirements [12].

Given the importance of P_i _to *Prochlorococcus*, perhaps it is surprising to find no significant correlation between ecotype distribution and P_i _concentration [13]. However, fluxes in P_i _transport within these regions are important considerations, which could help to explain the discrepancy. Nevertheless the observation of a large number of known P_i _acquisition genes in some LL ecotypes (i.e. MIT9313 and NATL2A), and not others (i.e. SS120) [14,15] is confusing. Indeed, P_i _acquisition genes are present in some HL strains (i.e. MED4) and not others (i.e. MIT9515) [14]. However it was recently observed that the prevalence of *Prochlorococcus *genes involved in acquisition of phosphate substrates were correlated with areas of low P_i _such as the Caribbean Sea and NW Mediterranean [16]. This conflict is likely resolved due to the presence of hypervariable genomic islands within *Prochlorococcus*, allowing for evolutionarily rapid niche adaptation [17]. Given this, it was hypothesised that the presence or absence of these genes could directly affect the protein content of cells when P_i _stressed, and as such directly affect the ability of a strain to acclimate to environmental P_i _fluctuations [16]. So the question arises, how effective are cells with and without these genes at acclimating to a shift in environmental P_i_? Indeed, the levels of mRNA transcripts of two strains, MED4 and MIT9313, which both contain the two component response regulation system *phoBR*, behaved quite differently to P_i _starvation [14].

To address this we selected three strains, MIT9312, NATL2A and SS120, each representative of an ecotype and a position within the water column (Table 1). MIT9312 is a HLII strain isolated at depth from the Gulf Stream. NATL2A is a LLI strain isolated from the North Atlantic which contains most of the P_i _acquisition genes found in MED4 and MIT9312, and yet is thought to experience both high and low light environments due to storm mixing events. SS120, originally isolated in the Sargasso Sea, does not possess *phoBR*, yet has two copies of the periplasmic phosphate binding protein, PstS. We took these three strains and allowed cells to acclimate to a significant reduction in environmental P_i _and investigated their respective protein contents.

**Table 1:** Details of the strains used in this study, as obtained from NCBI and CCMP.

Strain and genome details								
			Genome	Protein	%GC	Chl b/a	Ecotypic	
Strain	Refseq	Reference	size (Mbp)	coding		ratio	clade	
MIT9312	NC_007577	[18]^a^	1.71	1810	31	0.34	HLII	
NATL2A	NC_007335	[19]	1.84	2162	35	0.97	LLI	
SS120	NC_005042	[20]	1.75	1883	36	1.41	LLII	
P acquisition mechanisms								
	PhoBR cluster	PtrA cluster	PhoA	PhoE	PstS cluster	ArsA cluster	ArsB cluster	ArsC cluster
MIT9312	√	x	√	√	√	√	x	√
NATL2A	√	√	√	√	√	√	x	√
SS120	x	√	x	x	(2)	x	x	√
Isolation details and culture conditions								
	Location	Depth	Isolated by	Date	Culture temp (°C)	Deposited in	Media	
MIT9312	Gulf stream	135 m	L. Moore	17/07/1993	22-26	CCMP	Pro99	
NATL2A	N. Atlantic	10 m	D. Scanlan	01/04/1990	18-22	CCMP	Pro99	
SS120	Sargasso Sea	120 m	S. Frankel & L West-Johnsrud	01/01/1991	18-22	CCMP	Pro99	

^a ^indicates that the genome sequence has been submitted, yet not cleared. Ticks in '*P acquisition mechanisms*' indicates presence of gene/cluster, and copies are in parentheses

## Results and discussion

### Overview

**Figure 1:**
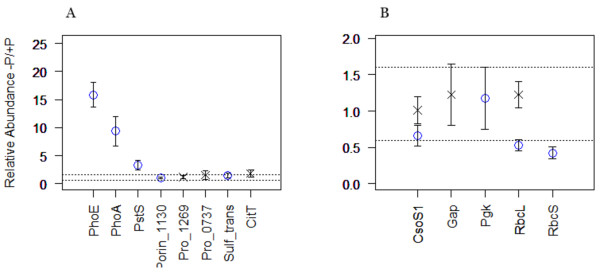
**Experimental growth curves of MIT9312, NATL2A and SS120 within P_i_replete (circles) and P_i_deplete (triangles) media**. Error bars represent one standard error. Growth rates (μ) are given with ± standard error.

The experimental growth data for each strain under P_i _replete and P_i _deplete cultures is shown in Figure 1. Logistic curve fitting and statistical analysis of the experimental growth data reveals no significant differences between the growth rates between P_i _replete and P_i _deplete cultures, with the exception of MIT9312 growth rates whereby P_i _replete growth was significantly greater than P_i _deplete growth (p < 0.05), as can be seen in Figure 1. It is important to consider the physiological status of the cells at the harvest point when considering protein relative abundances. Importantly, growth analysis shows that both MIT9312 and SS120 were in late exponential/early stationary phase at harvest, whilst NATL2A was in mid exponential phase. As the point of harvest differs for NATL2A, it would be difficult to directly compare the protein complement of NATL2A cells to either MIT9312 or SS120. Given this, the results for NATL2A will be discussed separately.

**Figure 2:**
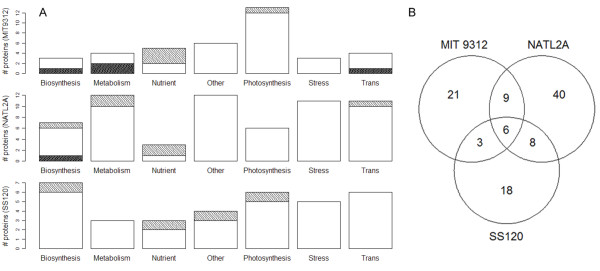
**(A) Distribution of proteins identified in all three strains within functional categories ('Trans' stands for 'Transcription, translation and stress')**. Light hatching represents proteins significantly more abundant than the control, and dark hatching represents proteins significantly less abundant than the control. (B) Venn diagram of all unique proteins specific to, or shared between, the strains.

Thirty eight, 63 and 34 proteins were identified with 2 or more peptides for strains MIT9312, NATL2A and SS120 respectively (Additional file 1: Table S1) with no false positives. An overview of the respective proteomes, through plotting theoretical values of isoelectric points (*pI*) against molecular weights (MW) reveal significant bias towards low *pI *values (Additional file 2: Figure S1), with no further correlation to MW, relative protein abundance, nor total peptide hits per protein (data not shown). This bias may be an artefact of the mass spectrometric analysis, where peptides are protonated directly before entry into the MS in order to assist flight and detection. As a consequence, naturally occurring proton-donor peptides may be preferentially selected. However, as there are no observable correlations between *pI *and peptide hits per protein, we can be confident that the intracellular protein abundances reported are directly reflective of the physiological status of the cells. Indeed, when interrogating the proportion of proteins with ≥ 50% of peptide hits, we see similarities between strains, such as the presence of RplL, RbcL and CsoS1 (Additional file 1: Table S1), however all three proteins have *pI *values < 7. Nevertheless, a high *pI *protein, PetH, is present in both MIT 9312 and SS120 samples. Also, identified proteins from all three strains are located evenly across the genomes, and are representative of most major functional groups such as central metabolism, photosynthesis, transcription and translation, biosynthesis and nutrient acquisition (Figure 2A). Of the 105 unique proteins identified, 6 were found in all three strains (Figure 2B). They are the ATP synthase subunits AtpA and AtpD, the PSII protein PsbO, the nitrogen regulatory protein GlnK, rubisco subunit RbcL, and the carboxysome shell protein CsoS1.

Using relative abundance cut-offs of 1.6 and 0.6 fold differences to represent increased or decreased relative abundances [21,22], 4 proteins were more abundant in MIT9312 and 4 were less abundant than the replete cultures. Within NATL2A, 6 proteins were more abundant and 1 was less abundant than the replete cultures. In SS120, 4 were more abundant and none were lower than the replete cultures (Figure 2A).

### Nutrient acquisition

**Figure 3:**
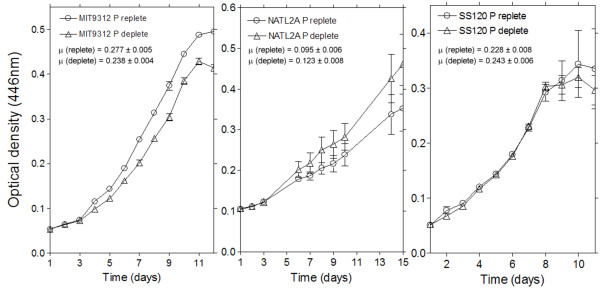
**Relative abundances of proteins associated with (A) nutrient acquisition and (B) central metabolism from MIT9312 (blue circles) and SS120 (black crosses)**. Dotted lines represent the abundance limits of 1.6 and 0.6. Error bars represent one standard error.

What is immediately apparent from our results is the differential abundance of P_i _acquisition proteins exhibited by all three strains to being grown in 10 μM P_i_. MIT 9312 demonstrates the greatest sensitivity to P_i_-deplete media, whereby the P_i _stress related porinPhoE is > 15-fold more abundant (Figure 3), the putative alkaline phosphatase PhoA appears to be > 9-fold greater, and the periplasmic P_i _binding protein PstS > 3 times more than the replete cultures. This result is directly in line with an earlier proteomic assay of P stress in a HL ecotype, MED4 [21], and closely reflective of microarray analyses of both MED4 and MIT9313 [14], *Synechococcucs *WH8102 [23], measured alkaline phosphatase activity of MIT9312 [15] and in line with observed responses within earlier P_i _depletion studies of other cyanobacteria [15,24-26].

Within NATL2A, PstS abundance is significantly greater within P_i_-deplete conditions, though with greater uncertainty (Additional file 1: Table S1). However neither PhoA nor PhoE was observed with mass spectrometry here, which is surprising as we showed previously that both PhoA and PhoE are greater in abundance alongside PstS in the high light ecotype MED4 [21], as is true with MIT9312 in this study. However, considering that NATL2A cells are in mid-exponential phase as opposed to early stationary phase this may indicate a progressive strategy of protein expression within the cells, however more work is needed to clarify this.

What was also unexpected, was the absence of any P_i _acquisition mechanisms (as reflected in observed peptide identifications) within SS120 cells (Additional file 1: Table S1), allied with no significant difference in growth rates between P_i_-replete and P_i_-deplete cultures (p > 0.05). SS120 is deficient in most P_i _acquisition genes [14,15], however it does have two copies of PstS, neither of which were present in our assay. At first glance, this result appears counter-intuitive, as a 'very' LL strain typically present *in vivo *within P_i_-replete environments would be expected to be adversely affected by a substantial decrease in P_i_. However, the absence of a *phoBR*regulon suggests that the strain is incapable of regulating a response to shifts in environmental concentrations of P_i _that are not immediately starvation inducing [27]. Curiously, this also infers that activation of the *phoBR *response mechanisms within MIT9312 and NATL2A were directly due to the mechanism's innate sensitivity to changing external P_i _concentrations. This suggests that the intensity of response is directly proportional to external P_i _concentration, coincidentally specific to each strain, and may be reflective of each strain's environmental niche and/or obligate cellular requirements.

### Photosynthesis, biosynthesis and central metabolism

**Figure 4:**
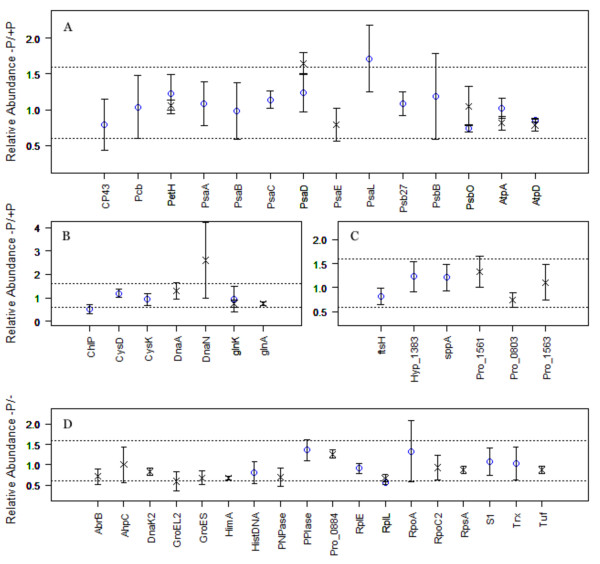
**Relative abundances of proteins associated with (A) photosynthesis, (B) biosynthesis, (C) uncategorised and (D) transcription, translation and stress from MIT9312 (blue circles) and SS120 (crosses)**. Dotted lines represent the abundance limits of 1.6 and 0.6. Error bars represent one standard error.

The exposure of all three strains to lower P_i _concentrations appears to have had little effect upon the photosynthetic machinery (Figure 4A and Additional file 1: Table S1). This is unusual, as P_i _depleted conditions have been previously noted to directly affect both photosystems in cyanobacteria [21,23,28]. In contrast, it is interesting to note that, for MIT9312, both Rubisco subunits (RbcL and RbcS) are noticeably lower in abundance (Figure 3B). This suggests that there is a progressive strategy within the cell when acclimating to lowered P_i_, whereby photosynthesis is initially dissociated from glycolysis, to then strategically break down the photosynthetic apparatus. This is a reasonable conclusion, considering a P_i_-induced organised break down of phycobilisomes has been previously observed in *Synechococcus *sp. PCC 7942 [29], chlorosis has been observed in thermophillic*Synechococcus *under P_i_-stress [28], and a strategic approach to a reduction in photosynthetic function has been hypothesised in MED4 [21]. Indeed, within WH8102 it appears that PSII was degraded before PSI, allowing continued cyclic photophosphorylation-based ATP generation to continue [23]. In this context, this could explain why an essential chlorophyll biosynthetic protein (ChlP) appears to be less abundant within P_i_-deplete MIT9312 cells (Figure 4B). However, it would be parsimonious to also expect a concurrent reduction in the light harvesting protein (Pcb) within P_i_-deplete MIT9312, which was noticed in MED4 [21], but there is no change. The reason for this is not clear.

When considering NATL2A solely, there appear to be a few subtle discrepancies in protein abundances between stressed and non-stressed cultures. Fumerase (FumC) is an enzyme associated with both the tricarboxylic acid (TCA) cycle and arginine/proline biosynthesis, and appears to be more abundant within NATL2A cells when P_i_-deplete (Additional file 1: Table S1). As NATL2A has an incomplete TCA cycle, it is safe to assume that its function within the cell is within arginine and proline metabolism. Also, the acyl carrier protein (AcpP) is an essential component of fatty acid biosynthesis, and is more abundant in P_i_-deplete NATL2A cells (Additional file 1: Table S1). Fatty acids are for the most part used within either fuel storage or membrane manufacture. However it may be misleading to arrive at the conclusion that this is a specific cellular response to lower P_i _concentrations. It is possibly a function of apparently slightly elevated (albeit not significant) growth within NATL2A P_i_-deplete cultures, and as such could reflect comparatively greater metabolic activity. Nevertheless, this explanation cannot immediately address the lower abundance of CobJ, a Precorrin-3B C17-methyltransferase region-containing protein (Additional file 1: Table S1), part of the aerobic vitamin B12 biosynthesis pathway within P_i_-stressed cells. However, B12 synthesis is a sub pathway offshoot from the main chlorophyll biosynthetic pathway, and as such may reflect a metabolic preference for chlorophyll production that, again, may be representative of faster growing populations.

An interesting observation is the abundance of CitT within P_i_-stressed SS120 cells (Figure 3A). This protein functions as a di/tricarboxylate transporter, which implies that the cells are scavenging lysed cellular material from the environment. That stressed SS120 cells appear to be preferentially acquiring tricarboxylic acid intermediates when growing in P_i_-deplete conditions, and not upregulatingPstS, is puzzling. However, it may indicate that this strain may be supplementing an affected glycolysis pathway through acquiring external carbon sources, and that this is more evidence that the cells response to an environmental stress is an iterative, evolving process. SS120 may simply have not initiated transcription of PstS in sufficiently detectable quantities. Indeed, even in starvation experiments *pstS*experession is far from an immediate response [14,23].

### Other proteins

An interesting observation is the presence of LuxR, the response regulatory family protein involved in quorum sensing within bacteria, in NATL2A cells (Additional file 1: Table S1). To our knowledge, this is the first instance of observing proteins putatively indicated in quorum sensing capability in any marine cyanobacteria. However, we were unable to locate any LuxI homologues, an essential protein required for effective quorum sensing, within NATL2A (data not shown). However LuxR is known to be a transcriptional regulator activated when cell concentrations of a particular trigger compound (usually *N*-(3-oxohexanoyl)-_L_-homoserine lactone, which is generated through the enzymatic functioning of LuxI) reach particular levels. As such, we speculate that the protein acts as a density-dependant transcriptional regulator, but for an unknown function, and through another trigger compound.

## Conclusions

*Prochlorococcus *are now widely considered to be evolutionarily adept at environmental niche domination, particularly within nutrient poor oligotrophic waters. The genus is typified by genomes characterised by hypervariable genomic islands [17], which are thought to contain genes obtained through phage-mediated horizontal gene transfer, and infer niche-specific advantages such as nutrient acquisition and phage resistance. Our results reinforce previous results concerning the importance of phosphate concentrations to specific strains, but also highlight the possibility of the cells employing a progressive acclimation strategy. It appears that *Prochlorococcus *strains evolutionarily adapted to life in a P_i_-deplete environment respond to phosphate fluctuations through a succession of cellular processes, such as the upregulation of P_i _acquisition mechanisms, a dissociation of photosynthesis from central metabolic pathways, and a staggered breakdown of the photosystems allowing prolonged photophosphorylated ATP generation. This progressive response allows the cell to react quickly to any subsequent increases in ambient P_i _concentrations. It is our hypothesis that HL strains are also particularly sensitive to changes in P_i_, and that ambient phosphate concentrations initiate a strong response regardless of being predominantly growth limited elsewhere.

We also note that our results strongly infer that the induction of P_i _acquisition mechanisms are concentration specific between strains, particularly considering the absence of any stress response of the LL strain SS120 compared to MIT9312 when grown from identical initial concentration levels.

## Methods

For a complete description of the Materials and Methods used please refer to the (Additional file 3: Material and Methods). In brief, however, biological triplicates of all three strains (MIT9312, NATL2A and SS120 (CCMP, Maine)) were grown under 2 separate conditions: P_i _replete (Pro99 media with 50 μM NaH_2_PO_4 _[30]) and P_i _deplete (Pro99 media with 10 μM NaH_2_PO_4_), and moderate white light intensities (30, 10 and 20 μE m^-2 ^s^-1 ^respectively), in a 13:11 h light:dark regime at 23°C.

For the proteomic analysis, the cells were harvested once measured optical densities reached 0.4 (after which populations had been observed to crash), and proteins were extracted from the three biological replicates for each phenotype [31]. 100 μg of protein from each replicate was then reduced, alkylated, digested and labelled with 8-plex iTRAQ reagents according to the manufacturer's (ABSciex, Framingham, MA) protocol. The labelled replicates were then pooled before primary strong cation exchange (SCX) fractionation [21]. Mass spectrometric analysis of the SCX fractions was performed with a QStar XL Hybrid ESI Quadrupole time-of-flight tandem mass spectrometer, ESI-qQ-TOF-MS/MS (Applied Biosystems; MDS Sciex, Concord, Ontario, Canada), coupled with an online capillary liquid chromatography system (Ultimate 3000, Dionex/LC Packings, The Netherlands) [21,22]. Preliminary data analysis, protein identification and quantitation were carried out using the PHENYX [Geneva Bioinformatics (GeneBio), Geneva, Switzerland] software platform.

## Competing interests

The authors declare that they have no competing interests.

## Authors' contributions

MAF designed the study, carried out the proteomics, analysed the data and drafted the manuscript. PCW and CAB conceived of the study and participated in its design. All authors read and approved the final manuscript.

## Supplementary Material

Additional file 1 **Table S1**. Full list of identified proteins and peptides for all 3 strains used in this study. Click here for file

Additional file 2 **Figure S1**. Virtual 2D gel representations of proteins identified from MIT9312 (top left), NATL2A (top right), and SS120 (bottom left). Click here for file

Additional file 3 **Materials and methods **[32-34]. Click here for file

## References

[B1] Ammerman JW Hood RR Case DA Cotner JB Phosphorus deficiency in the Atlantic: An emerging paradigm in oceanography Eos Trans Am Geophys Union 2003 84 165 170

[B2] Thingstad TF Krom MD Mantoura RF Nature of phosphorus limitation in the ultraoligotrophic eastern Mediterranean Science 2005 309 1068 1071 10.1126/science.1112632 16099984

[B3] Thingstad TF Zweifel UL Rassoulzadegan F P limitation of heterotrophic bacteria and phytoplankton in the northwest Mediterranean Limnol Oceanogr 1998 43 88 94 10.4319/lo.1998.43.1.0088

[B4] Chisholm SW Olson RJ Zettler ER Goericke R Waterbury JB Welschmeyer NA A novel free-living prochlorophyte abundant in the oceanic euphotic zone Nature 1988 334 340 343 10.1038/334340a0

[B5] Partensky F Hess WR Vaulot D *Prochlorococcus*, a marine photosynthetic prokaryote of global significance Microbiol Mol Biol Rev 1999 63 106 127 10066832 10.1128/mmbr.63.1.106-127.1999 PMC98958

[B6] Moore LR Chisholm SW Photophysiology of the marine cyanobacterium *Prochlorococcus*: ecotypic differences among cultured isolates Limnol Oceanogr 1999 44 628 638 10.4319/lo.1999.44.3.0628

[B7] Moore LR Rocap G Chisholm SW Physiology and molecular phylogeny of coexisting *Prochlorococcus *ecotypes Nature 1998 393 464 467 10.1038/30965 9624000

[B8] Rocap G Distel DL Waterbury JB Chisholm SW Resolution of *Prochlorococcus *and *Synechococcus *ecotypes by using 16S-23S ribosomal DNA internal transcribed spacer sequences Appl Environ Microbiol 2002 68 1180 1191 10.1128/AEM.68.3.1180-1191.2002 11872466 PMC123739

[B9] Partensky F Garczarek L *Prochlorococcus*: Advantages and Limits of Minimalism Ann Rev Marine Sci 2009 2 305 331 10.1146/annurev-marine-120308-081034 21141667

[B10] Coleman ML Chisholm SW Ecosystem-specific selection pressures revealed through comparative population genomics ProcNatlAcadSci 2010 107 18634 18639 10.1073/pnas.1009480107 PMC2972931 20937887

[B11] Dufresne A Garczarek L Partensky F Accelerated evolution associated with genome reduction in a free-living prokaryote Genome Biol 2005 6 R14 10.1186/gb-2005-6-2-r14 15693943 PMC551534

[B12] Van Mooy BA Rocap G Fredricks HF Evans CT Devol AH Sulfolipids dramatically decrease phosphorus demand by picocyanobacteria in oligotrophic marine environments Proc Natl Acad Sci USA 2006 103 8607 8612 10.1073/pnas.0600540103 16731626 PMC1482627

[B13] Johnson ZI Zinser ER Coe A McNulty NP Woodward EM Chisholm SW Niche partitioning among *Prochlorococcus* ecotypes along ocean-scale environmental gradients Science 2006 311 1737 1740 10.1126/science.1118052 16556835

[B14] Martiny AC Coleman ML Chisholm SW Phosphate acquisition genes in *Prochlorococcus *ecotypes: evidence for genome-wide adaptation Proc Natl Acad Sci USA 2006 103 12552 12557 10.1073/pnas.0601301103 16895994 PMC1567916

[B15] Moore LR Ostrowski M Scanlan DJ Feren K Sweetsir T Ecotypic variation in phosphorus-acquisition mechanisms within marine picocyanobacteria AquatMicrobEcol 2005 39 257 269

[B16] Martiny AC Huang Y Li W Occurrence of phosphate acquisition genes in *Prochlorococcus *cells from different ocean regions Environ Microbiol 2009 11 1340 1347 10.1111/j.1462-2920.2009.01860.x 19187282

[B17] Coleman ML Sullivan MB Martiny AC Steglich C Barry K Delong EF Chisholm SW Genomic islands and the ecology and evolution of *Prochlorococcus* Science 2006 311 1768 1770 10.1126/science.1122050 16556843

[B18] Copeland A Lucas S Lapidus A Barry K Detter JC Hammon N Israni S Pitluck S Thiel J Schmutz J Larimer F Land M Kyrpides N Lykidis A Richardson P Complete sequence of *Prochlorococcus marinus* str MIT 2005 9312 Unpublished

[B19] Kettler GC Martiny AC Huang K Zucker J Coleman ML Rodrigue S Chen F Lapidus A Ferriera S Johnson J Patterns and implications of gene gain and loss in the evolution of *Prochlorococcus* PLoS Genet 2007 3 e231 10.1371/journal.pgen.0030231 18159947 PMC2151091

[B20] Dufresne A Salanoubat M Partensky F Artiguenave F Axmann IM Barbe V Duprat S Galperin MY Koonin EV Le Gall F Makarova KS Ostrowski M Oztas S Robert C Rogozin IB Scanlan DJ Tandeau de Marsac N Weissenbach J Wincker P Wolf YI Hess WR Genome sequence of the cyanobacterium *Prochlorococcus marinus* SS120, a nearly minimal oxyphototrophic genome Proc Natl Acad Sci USA 2003 100 17 10020 10025 10.1073/pnas.1733211100 12917486 PMC187748

[B21] Fuszard MA Wright PC Biggs CA Cellular acclimation strategies of a minimal picocyanobacterium to phosphate stress FEMS MicrobiolLett 2010 306 127 134 10.1111/j.1574-6968.2010.01942.x 20370833

[B22] Pandhal J Wright PC Biggs CA A quantitative proteomic analysis of light adaptation in a globally significant marine cyanobacterium *Prochlorococcus marinus *MED4 J Proteome Res 2007 6 996 1005 10.1021/pr060460c 17298086

[B23] Tetu SG Brahamsha B Johnson DA Tai V Phillippy K Palenik B Paulsen IT Microarray analysis of phosphate regulation in the marine cyanobacterium *Synechococcus *sp. WH8102 ISME J 2009 3 835 849 10.1038/ismej.2009.31 19340084

[B24] Huber AL Hamel KS Phosphatase activities in relation to phosphorus nutrition in *Nodularia spumigen* (Cyanobacteriaceae) Hydrobiologia 1985 123 81 88 10.1007/BF00006617

[B25] Natesan R Shanmugasundaram S Extracellular phosphate solubilization by the cyanobacterium *Anabaena *ARM310 J Biosci 1989 14 203 208 10.1007/BF02716680

[B26] Scanlan DJ Mann NH Carr NG The response of the picoplanktonic marine cyanobacterium *Synechococcus *species WH7803 to phosphate starvation involves a protein homologous to the periplasmic phosphate-binding protein of *Escherichia coli* Mol Microbiol 1993 10 181 191 10.1111/j.1365-2958.1993.tb00914.x 7968514

[B27] Scanlan DJ West NJ Molecular ecology of the marine cyanobacterial genera *Prochlorococcus *and *Synechococcus* FEMS Microbiol Ecol 2002 40 1 12 10.1111/j.1574-6941.2002.tb00930.x 19709205

[B28] Adams MM Gomez-Garcia MR Grossman AR Bhaya D Phosphorus Deprivation Responses and Phosphonate Utilization in a Thermophilic *Synechococcus *sp. from Microbial Mats J Bacteriol 2008 190 8171 8184 10.1128/JB.01011-08 18931115 PMC2593230

[B29] Collier JL Grossman AR Chlorosis induced by nutrient deprivation in *Synechococcus *sp. strain PCC 7942: not all bleaching is the same J Bacteriol 1992 174 4718 4726 1624459 10.1128/jb.174.14.4718-4726.1992 PMC206268

[B30] Moore LR Post AF Rocap G Chisholm SW Utilization of Different Nitrogen Sources by the Marine Cyanobacteria *Prochlorococcus *and *Synechococcus* LimnolOceanogr 2002 47 989 996

[B31] Meijer EA Wijffels RH Development of a Fast, Reproducible and Effective Method for the Extraction and Quantification of Proteins of Micro-algae Biotechnol Tech 1998 12 353 358 10.1023/A:1008814128995

[B32] Moore LR Goericke R Chisholm SW Comparative physiology of *Synechococcus *and *Prochlorococcus*: Influence of light and temperature on growth, pigments, fluorescence and absorptive properties Mar Ecol Prog Ser 1995 116 259 276

[B33] Ow SY Noirel J Cardona T Taton A Lindblad P Stensjo K Wright PC Quantitative Overview of N2 Fixation in *Nostoc punctiforme *ATCC 29133 through Cellular Enrichments and iTRAQ Shotgun Proteomics Journal Proteome Research 2009 8 187 198 10.1021/pr800285v 19012430

[B34] Team RDC R: A Language and Environment for Statistical Computing 2011 R Foundation for Statistical Computing: Vienna, Austria

