# Supplementary material for: Comparative quantitative proteomics of prochlorococcus ecotypes to a decrease in environmental phosphate concentrations
Source: Aquat Biosyst. 2012 Mar 19;8:7. doi: 10.1186/2046-9063-8-7 (PMC3349580; doi:10.1186/2046-9063-8-7)
Supplement: Additional file 1 — Table S1. Full list of identified proteins and peptides for all 3 strains used in this study. [file 2046-9063-8-7-S1.DOC]

**Supplementary Material 1: Materials and methods**

*Growth conditions and analysis*

Non-axenic *Prochlorococcus* strains MIT 9312, NATL2A and SS120 (Table 1) were grown at a constant 21˚C within sterile 75 cm^2^ Nunc ® EasYFlasks ™ (Thermo Fisher Scientific, Rochester, NY) in a Sanyo MLR-350H environmental chamber under cool white fluorescent lights. The strains were grown under a 13:11 hour light:dark regime, and monitored in late afternoon, before the onset of the dark period. All cultures were initially grown within phosphate replete Pro99 culture media (Moo*re, et a*l., 2002) for at least 3 transfers before the start of the experiment. The experimental conditions comprised the individual strains being grown at specific light intensities: MIT 9312, SS120 and NATL2A were grown with intensities of 30, 20, and 10 μ E m^-2^ s^-1^ respectively, at 23˚C. Light intensity was measured using a Loggerhead 2100 light probe (Biospherical instruments, San Diego, CA). These intensities were chosen due to the observation of robust growth during acclimation. Although the selection of light intensities does not directly reflect their respective positions within the water column, the region of intensities between 10-30 μE m^-2^ s^-1^ was selected to avoid subjecting any of the strains to high light stress, and to keep the conditions as close to each other as possible.

To assess each strains response to a phosphate limiting environment, cells were grown in triplicate within replete (50 μM NaH_2_PO_4_ added to) and deplete (10 μM NaH_2_PO_4_) media, and growth was monitored using an UltraSpec 2100 Pro spectrophotometer (Amersham Biosciences, Buckinhamshire, UK) at 446nm (which relates to the Chl *a_2_* absorbance peak (Moo*re, et a*l., 1995)). 50 μM is the concentration of phosphate within stock media for the growth of the strains. The purpose of the study is to observe any significant shifts in protein concentrations that would represent an acclimation strategy. Therefore, 10μM was selected as this would represent a significant reduction in external concentrations that were not starvation inducing.

MIT 9312 and SS120 cultures were harvested upon the initiation of stationary phase, and NATL2A cultures were harvested in mid-exponential phase. This was due to observations of stock cultures collapsing after an optical density of 0.4 had been reached (Figure 1).

To analyse the comparative growth of all 3 strains, growth rates were analysed by fitting exponential growth models to sections of the experimental growth data and accepting values which afforded the greatest fit (R^2^ >0.99). Statistical analysis was performed using the R package [3].

*Protein extraction and quantitation*

All cultures were initially centrifuged at 21 000g for 20 minutes at 4˚C (Heraeus Multifuge 3 S-R, Germany), and resuspended in 500 nM triethylammonium bicarbonate (TEAB) buffer. Proteins were extracted through mechanical cracking using liquid nitrogen and further centrifuged to separate the water soluble lysis proteins from the membrane fraction. Initial protein concentrations were assayed with RC DC reagents (Bio-Rad Laboratories, Hercules, CA) as per the manufacturers instructions.

Approximately 100 μg of protein from each sample was placed in PCR clean LoBind microcentrifuge tubes (Eppendorf, Cambridgeshire, UK), then reduced, alkylated and digested.

*iTRAQ protein quantitation*

The resultant peptide digests were then labelled using iTRAQ chemical reagents, whereby the moieties 113,114 and 115 were added to the phosphate replete control replicates, and the moieties 116, 117 and 118 were added to the phosphate deplete replicates, as per the manufacturer’s instructions. Primary peptide fractionation was carried out through strong cation exchange (SCX) on a BioLC HPLC unit (Dionex, Surrey, UK) with a Poly SULFOETHYL™ A column (PolyLC, Columbia, MD, USA). The column used 5μm particle size, and had dimensions of 200 mm length with an internal diameter of 2.1 mm and a 200 Å pore size. The buffers used were Buffer A (10 mM KH_2_PO_4_ with 20% HPLC grade Acetonitrile (ACN) at pH 2.5) and Buffer B (10 mM KH_2_PO_4_ with 20% HPLC grade Acetonitrile (ACN) and 500 mM KCl at pH3) with a gradient of 0% B for 5 minutes, 0-40% B for 30 minutes, 40-100% B for 10 minutes, 100% B for 5 minutes, and 0% B for 5 minutes. Before fractionation the digests were dried in a vacuum centrifuge and resuspended in 200 μl Buffer A, and two injections were completed before initiation of the gradient. Fractions were collected each minute for the duration of the run. Fractions were selected for subsequent MS/MS analysis according to SCX chromatogram intensity.

Mass spectrometric analysis was performed on a QStar XL Hybrid ESI Quadrupole time-of-flight tandem mass spectrometer, ESI-qQ-TOF MS/MS (Applied Biosystems, Framingham, MA; MDS-Sciex, Concord, Ontario, Canada), coupled with an online capillary liquid chromatography (LC) system (Applied Biosystems, Framingham, MA), with a PepMap C-18 RP column (LC Packings) set to a constant flow rate of 0.3 μl min^-1^. The LC-MS/MS buffers were Buffer A (3% ACN with 0,1% Formic acid (FA)), and Buffer B (97%ACN with 0.1% FA). Selected fractions were dried and resuspended in 10 μl of Buffer A prior to analysis. To prevent salts, and other contaminants entering the mass spectrometer, a C-18 column clean up (The Nest Group, Inc. Southborough, MA) of each fraction was performed. Cleaned samples were then injected into the online nanoflow LC-MS/MS unit where the buffers were Buffer A (3% ACN with 0,1% Formic acid (FA)), and Buffer B (97%ACN with 0.1% FA). The gradient applied for the second dimension of chromatography was 3% Buffer B for 3 minutes, 3 – 40% Buffer B for 70 minutes, 90% Buffer B for 5 minutes, and 3% Buffer B for 7 minutes. The process of electrospray ionization (ESI) can be set to either a positive ion mode (the addition of a proton) or negative ion mode (the removal of an electron) in order to charge the analyte before entry into the MS. For this analysis the data acquisition protocol in the mass spectrometer was set to positive ion mode, and the addition of Formic acid to the buffers was used in order to promote protonation. Peptides were selected by the mass spectrometer for further tandem MS/MS due to observed charge states of +2 and +3.

Preliminary data analysis, peptide identification and quantitation was carried out using the Phenyx (Geneva Bioinformatics (GeneBio), Geneva, Switzerland) software, with the parameter settings: Trypsin enzyme, cleavage mode: normal and one missed cleavage; peptide thresholds: both p ≤ 10^-4^ with a score ≥ 5, and p ≤ 10^-6^ with a score ≥ 7. Protein identification was carried out through comparing sequence hits to the annotated FASTA genome sequences of each strain, and the non-redundant database (<http://www.ncbi.nlm.nih.gov/>). The Q scores are the iTRAQ-derived abundance values, which were calculated using Mathematica v7.0 (Wolfram Research, Oxfordshire UK) based on principles derived by Ow et al (2009). Briefly, the calculations involved the geometric average of intensities, the median correction of protein quants and the isotopic correction of reporter intensities.

**References:**

[1] Moore LR, Goericke R & Chisholm SW (1995) Comparative physiology of Synechococcus and Prochlorococcus: Influence of light and temperature on growth, pigments, fluorescence and absorptive properties. *Marine Ecology Progress Series* **116**: 259-276.

[2] Moore LR, Post AF, Rocap G & Chisholm SW (2002) Utilization of Different Nitrogen Sources by the Marine Cyanobacteria *Prochlorococcus* and *Synechococcus*. *Limnol Oceanogr* **47**: 989-996.

[3] Team RDC (2011) R: A language and environment for statistical computing. R Foundation for Statistical Computing, Vienna, Austria.
