# Supplementary material for: Comparative quantitative proteomics of prochlorococcus ecotypes to a decrease in environmental phosphate concentrations
Source: Aquat Biosyst. 2012 Mar 19;8:7. doi: 10.1186/2046-9063-8-7 (PMC3349580; doi:10.1186/2046-9063-8-7)
Supplement: Additional file 2 — Figure S1. Virtual 2D gel representations of proteins identified from MIT9312 (top left), NATL2A (top right), and SS120 (bottom left). [file 2046-9063-8-7-S2.DOC]

**Supplementary figure**


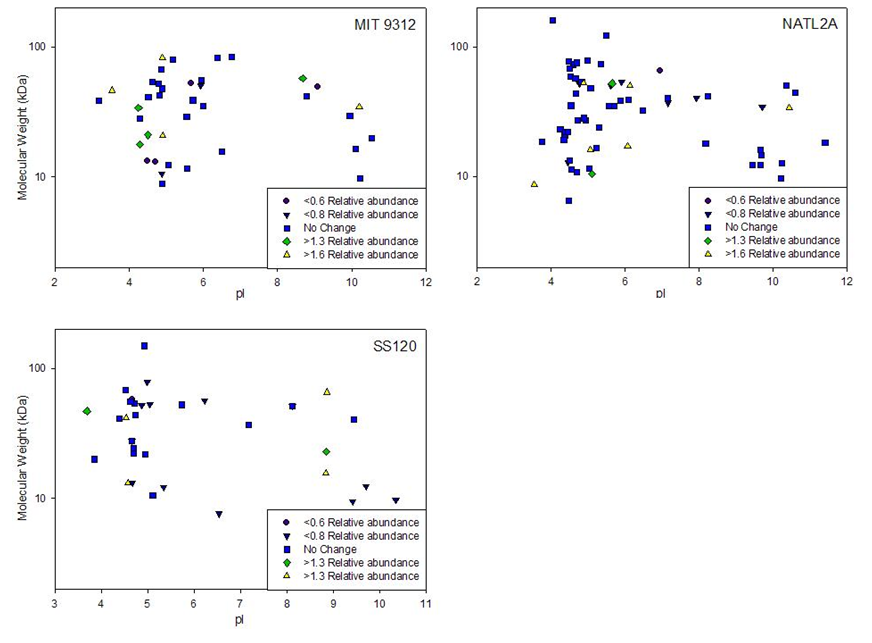


**Figure S1.** Virtual 2D gel representations of proteins identified from MIT9312 (top left), NATL2A (top right), and SS120 (bottom left).
